# Higher prevalence of sacbrood virus in *Apis mellifera* (Hymenoptera: Apidae) colonies after pollinating highbush blueberries

**DOI:** 10.1093/jee/toae119

**Published:** 2024-06-15

**Authors:** Alison McAfee, Sarah K French, Sydney B Wizenberg, Laura R Newburn, Nadejda Tsvetkov, Heather Higo, Julia Common, Stephen F Pernal, Pierre Giovenazzo, Shelley E Hoover, Ernesto Guzman-Novoa, Robert W Currie, Patricia Wolf Veiga, Ida M Conflitti, Mateus Pepinelli, Lan Tran, Amro Zayed, M Marta Guarna, Leonard J Foster

**Affiliations:** Department of Biochemistry and Molecular Biology, Michael Smith Laboratories, University of British Columbia, Vancouver, BC V6T1Z4, Canada; Department of Applied Ecology, North Carolina State University, Raleigh, NC 27695, USA; Department of Biology, York University, Toronto, ON M3J 1P3, Canada; Department of Biology, York University, Toronto, ON M3J 1P3, Canada; Department of Biology, York University, Toronto, ON M3J 1P3, Canada; Department of Biochemistry and Molecular Biology, Michael Smith Laboratories, University of British Columbia, Vancouver, BC V6T1Z4, Canada; Department of Biochemistry and Molecular Biology, Michael Smith Laboratories, University of British Columbia, Vancouver, BC V6T1Z4, Canada; Department of Biochemistry and Molecular Biology, Michael Smith Laboratories, University of British Columbia, Vancouver, BC V6T1Z4, Canada; Beaverlodge Research Farm, Agriculture and Agri-Food Canada, Beaverlodge, AB T0H 0C0, Canada; Département de Biologie, Université Laval, Ville de Québec, QC G1V 0A6, Canada; Department of Biological Sciences, University of Lethbridge, Lethbridge, AB T1K 3M4, Canada; School of Environmental Sciences, University of Guelph, Guelph, ON N1G 2W1, Canada; Department of Entomology, University of Manitoba, Winnipeg, MB R3T 2N2, Canada; National Bee Diagnostic Centre, Northwestern Polytechnic, Beaverlodge, AB T0H 0C0, Canada; Department of Biology, York University, Toronto, ON M3J 1P3, Canada; Department of Biology, York University, Toronto, ON M3J 1P3, Canada; Beaverlodge Research Farm, Agriculture and Agri-Food Canada, Beaverlodge, AB T0H 0C0, Canada; Department of Biology, York University, Toronto, ON M3J 1P3, Canada; Department of Biochemistry and Molecular Biology, Michael Smith Laboratories, University of British Columbia, Vancouver, BC V6T1Z4, Canada; Beaverlodge Research Farm, Agriculture and Agri-Food Canada, Beaverlodge, AB T0H 0C0, Canada; Department of Biochemistry and Molecular Biology, Michael Smith Laboratories, University of British Columbia, Vancouver, BC V6T1Z4, Canada

**Keywords:** honey bees, blueberries, EFB, pathogens, pollination

## Abstract

Highbush blueberry pollination depends on managed honey bees (*Apis mellifera* L.) for adequate fruit sets; however, beekeepers have raised concerns about the poor health of colonies after pollinating this crop. Postulated causes include agrochemical exposure, nutritional deficits, and interactions with parasites and pathogens, particularly *Melisococcus plutonius* [(ex. White) Bailey and Collins, Lactobacillales: Enterococcaceae], the causal agent of European foulbrood disease, but other pathogens could be involved. To broadly investigate common honey bee pathogens in relation to blueberry pollination, we sampled adult honey bees from colonies at time points corresponding to before (t1), during (t2), at the end (t3), and after (t4) highbush blueberry pollination in British Columbia, Canada, across 2 years (2020 and 2021). Nine viruses, as well as *M. plutonius*, *Vairimorpha ceranae,* and *V. apis* [Tokarev et al., Microsporidia: Nosematidae; formerly *Nosema ceranae* (Fries et al.) and *N. apis* (Zander)], were detected by PCR and compared among colonies located near and far from blueberry fields. We found a significant interactive effect of time and blueberry proximity on the multivariate pathogen community, mainly due to differences at t4 (corresponding to ~6 wk after the beginning of the pollination period). Post hoc comparisons of pathogens in near and far groups at t4 showed that detections of sacbrood virus (SBV), which was significantly higher in the near group, not *M. plutonius*, was the primary driver. Further research is needed to determine if the association of SBV with highbush blueberry pollination is contributing to the health decline that beekeepers observe after pollinating this crop.

## Introduction

Highbush blueberry [*Vaccinium corymbosum* L. (Ericales: Ericaceae)] is a major crop in Canada and the United States (Protzman 2021, Agriculture and Agri-Food Canada 2023) and fruit production is enhanced by insect pollination, which increases fruit weight by approximately 75% (Eeraerts et al. 2023). Many native bee species, especially those capable of “buzz-pollination” (vibrational disturbance of pollen), are more efficient pollinators of blueberry flowers than honey bees (*Apis mellifera*) (Rogers et al. 2013, 2014, Hoffman et al. 2018, Cortés-Rivas et al. 2023). However, honey bees, which are not native to the Americas, can be stocked at high densities and moved to specific locations, making them a common commercial pollinator (Isaacs and Kirk 2010, Gibbs et al. 2016, Hoffman et al. 2018). British Columbia accounts for 95% of Canada’s total highbush blueberry production, with particularly high densities of blueberry fields occurring in the Fraser Valley (Agriculture and Agri-Food Canada 2023).

Blueberry pollination contracts are an important source of income for beekeepers (Bixby et al. 2023). However, some beekeepers and researchers have raised concerns that colony health deteriorates after engaging in highbush blueberry pollination (Wardell 1982, Higo et al. 2019, Thebeau et al. 2022, 2023). Several sources have postulated that European foulbrood (EFB, caused by *Melissococcus plutonius*), exposure to fungicides, nutritional deficits, and their interactions could be the underlying causes of poor colony health outcomes perceived to be associated with highbush blueberry pollination (Wardell 1982, Graham et al. 2021, 2022, Thebeau et al. 2023). Notably, these poor health outcomes have not yet been empirically documented as being distinct from seasonal trends that may overlap with the blueberry pollination period.

However, fungicides and their adjuvants (nonactive ingredients that enhance pesticide performance, such as surfactants) can increase honey bee susceptibility to pathogens, such as *Vairimorpha ceranae* (a microsporidian parasite) (Pettis et al. 2013, Tokarev et al. 2020), viruses (Degrandi-Hoffman et al. 2015, Fine et al. 2017, O’Neal et al. 2019), and, in some cases, *M. plutonius* (Thebeau et al. 2023). Fungicide applications to blueberry fields are sometimes necessary to control diseases that devalue the berries or damage the plants, such as anthracnose, botrytis, mummyberry, and root rot (Agriculture and Agri-Food Canada 2007). Honey bees can be exposed to fungicides and other agrochemicals while foraging on blueberry and nonblueberry plants (Pettis et al. 2013, Graham et al. 2022), and these interactions between fungicides and pathogens broadly highlight the utility of analyzing multivariate pathogen data in field trials.

EFB is a bacterial disease affecting honey bee larvae (Forsgren 2010, Lewkowski and Erler 2019). It is thought to be a highly prevalent but opportunistic pathogen with symptoms tending to appear during spring, sometimes in association with additional stressors (Wardell 1982, Bailey and Ball 1991, Fünfhaus et al. 2018, Grant et al. 2021) or changes in colony demography in response to nectar flows (Bailey 1960, Milbrath 2021). EFB presentation sometimes coincides with blueberry pollination (Gregoris et al. 2019, Grant et al. 2021), but a recent controlled study in Michigan calls that association into question (Fowler et al. 2023). In addition, while deformed wing virus loads do not influence a colony’s likelihood of developing EFB (Fowler et al. 2023) relationships with other diseases have not been fully explored.

Here, we conducted an experiment evaluating 12 pathogens, which are among those most commonly observed in honey bee colonies (Evans and Schwarz 2011, Borba et al. 2022), in colonies at different proximities to highbush blueberry fields in British Columbia. Colonies were placed near highbush blueberry fields (“near” sites) or at least 1.3 km away from blueberry fields (“far” sites) and sampled over time, starting immediately before the highbush blueberry pollination period and ending approximately 2 wk after the pollination period ended. This design allowed us to test the hypothesis that pathogen profiles in colonies placed near and far from blueberries would differ over time and that *M. plutonius* detections, in particular, would be higher in colonies placed near blueberries after pollination concluded.

## Materials and Methods

### Honey Bee Colonies and Field Sites

The honey bees used in this study consisted of 40 colonies in 2020 and 40 different colonies in 2021, some of which (“near” colonies, described below) have been reported elsewhere (French et al. 2024), and all of which were managed by the research team in both years. In 2020, the experimental colonies were produced on-site from overwintered colonies headed by queens overwintered in British Columbia, Canada. The donor colonies were treated for *Varroa* mites in early April with Formic Pro according to manufacturer’s instructions. In 2021, experimental colonies were produced from nucleus colonies headed by locally mated, overwintered BC queens and allowed to grow until the beginning of the experiment. Each colony was initially housed in a single-box, deep Langstroth hive, with additional boxes added as needed to suppress swarming as the population expanded over time. Colony sizes followed conventions used for blueberry pollination units (queenright, minimum of 4 frames of brood, 8 frames of adult bees), and colonies received no supplemental feeding or medications during the course of the experiment.

**Fig. 1.**
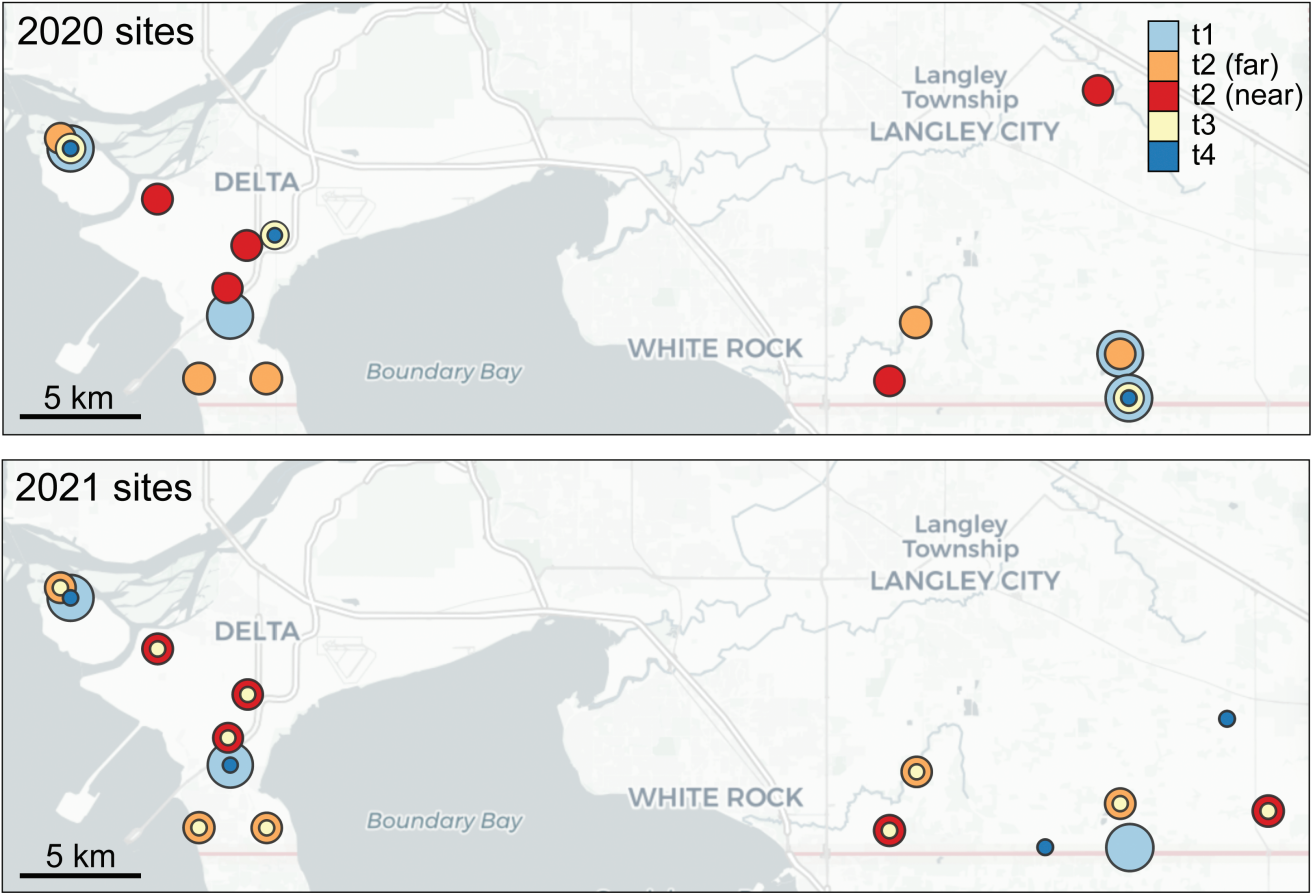
Experimental sites. *N* = 10 pooled samples (5 sites near highbush blueberries and 5 sites far from highbush blueberries), representing 40 colonies, were evaluated in 2020. T1 (at holding yards prior to moving into sites near or far from blueberry fields), t2 (at near or far yards in the middle of the pollination period), t3 (immediately before or immediately after moving to post-pollination yards at the end of the polilnation period), and t4 (at post-polilnation yards) samples were taken at approximately 2-wk intervals. Map base layer—Copyright: OpenStreetMap and CARTO, licensed under the Open Data Commons Open Database License (ODbL) by the OpenStreetMap Foundation (OSMF), (CC BY-SA 2.0).

Each colony was sampled at 4 time points and 3 different locations: before pollination (t1, holding yard), during pollination (t2, at yards near or far from blueberry fields, referred to as “near” and “far” yards, respectively), at the end of pollination (t3, same yards as t2 or immediately after moving out), and after the end of pollination (t4, postpollination yard). A map of site locations can be found in Fig. 1. Each time point was approximately 2 wk apart (see Table 1 for exact dates). Samples at t1 holding yards were taken immediately before moving colonies to their respective near and far site types, t2 samples were taken at peak blueberry bloom, t3 samples were taken at the end of the pollination period (immediately before moving to postpollination yards in 2021 and immediately after moving to postpollination yards in 2020), and t4 samples were taken at postpollination yards, 2 wk after the end of the pollination period.

**Table 1.** Sampling dates for the highbush blueberry field experiment

Year	Activity	Sampling date	Days from latest t1
2020	t1 sampling	April 27–28	0
	Move to blueberry near/far yards	April 29	1
	t2 sampling	May 11	14
	Move to postpollination yards	May 25	27
	t3 sampling	May 26	28
	t4 sampling	June 11	44
2021	t1 sampling	April 23	0
	Move to blueberry near/far yards	April 24-25	1–2
	t2 sampling	May 10	17
	t3 sampling	May 21	28
	Move to postpollination yards	May 22–23	29–30
	t4 sampling	June 2	40

Sites for the pollination yards were categorized into 2 groups (“near” and “far” from blueberry fields). These sites were chosen such that sites were >3 km from one another, with the exception of 2 near-blueberry sites, which were 2 km apart and a near and far site within 2.7 km of each other. In each year, pollination yards were composed of 5 near (4 of which were used in both years) and 5 far sites (all of which were used in both years) (Fig. 1).

Sites were initially chosen with the goal of having near sites located immediately adjacent to established blueberry plots and for far sites to be located at least 1.5 km away from established blueberry plots, but this was not always achievable. In the region where this study was conducted (the Fraser Valley of British Columbia), far sites >1.5 km from blueberry fields were exceedingly difficult to identify, and as a result, 4 out of 10 far sites still had some blueberry cropland occurring within 1.5 km of the site, but not closer than 1.3 km. Average foraging distances from colonies reported in the literature are variable, ranging from 0.4 to 0.6 km (Schneider and McNally 1993), 0.5–1.1 km (Waddington et al. 1994), 1.1–1.4 km (Schneider and Hall 1997), 1.2 km (Schneider 1989), 2.3 km (Visscher and Seeley 1982), and 5.5 km (Beekman and Ratnieks 2000). However, foraging ranges may be reduced to even less than 1 km in areas of intense agricultural production, where floral resources are high (Couvillon et al. 2014, Balfour and Ratnieks 2017). In light of the above-cited data on foraging distances in agricultural contexts, we consider the “far” threshold of >1.3 km away from blueberries as satisfactory and appropriate in this context.

We determined blueberry proximity and blueberry field coverage by inspecting each site in each year using a combination of Google Earth, additional site visits, and the World Imagery Wayback archive (https://livingatlas.arcgis.com/wayback). Blueberry plots are identifiable in aerial photographs by the following features: (i) wide row spacing (1.8–2.4 m), (ii) irregular globular shape of bushes, (iii) winter defoliation, and (iv) perennial establishment. Raspberry fields can appear similar but have distinct cane-shaped trellising. Ambiguous plots were confirmed in some cases by site visits and, where available, Google Maps “streetview.” Based on these assessments, our near and far sites were an average of 53 m and 2,352 m from the nearest blueberry field, respectively, and total blueberry coverage within a 1.5 km radius averaged at 0.1% and 11.4%, respectively. See Table 2 for GPS location, blueberry coverage, and distance information for each site.

**Table 2.** Description of pollination yards

Year	Site	Latitude	Longitude	Blueberry area (%)	Distance to nearest blueberry plot (m)
2020	HBB01 Far	49.0118	−123.0903	0.0	3,625
2020	HBB01 Near	49.0609	−123.0633	16.0	22
2020	HBB02 Far	49.0121	−123.0519	0.0	3,321
2020	HBB02 Near	49.0454	−123.0747	19.3	3
2020	HBB03 Far	49.1012	−123.1697	0.1	1,400
2020	HBB03 Near	49.0780	−123.1142	10.2	80
2020	HBB04 Far	49.0325	−122.6844	0.0	2,104
2020	HBB04 Near	49.0105	−122.6997	1.8	138
2020	HBB05 Far	49.0208	−122.5693	0.6	1,309
2020	HBB05 Near	49.1187	−122.5820	7.6	21
2021	HBB06 Far	49.0208	−122.5693	0.6	1,309
2021	HBB06 Near	49.0184	−122.4853	12.9	22
2021	HBB07 Far	49.0325	−122.6844	0.0	2,104
2021	HBB07 Near	49.0105	−122.6997	1.8	138
2021	HBB08 Far	49.1012	−123.1697	0.1	1,400
2021	HBB08 Near	49.0780	−123.1142	9.0	80
2021	HBB09 Far	49.0118	−123.0903	0.0	3,625
2021	HBB09 Near	49.0609	−123.0633	16.0	22
2021	HBB10 Far	49.0121	−123.0519	0.0	3,321
2021	HBB10 Near	49.0454	−123.0747	19.3	3

**Fig. 2.**
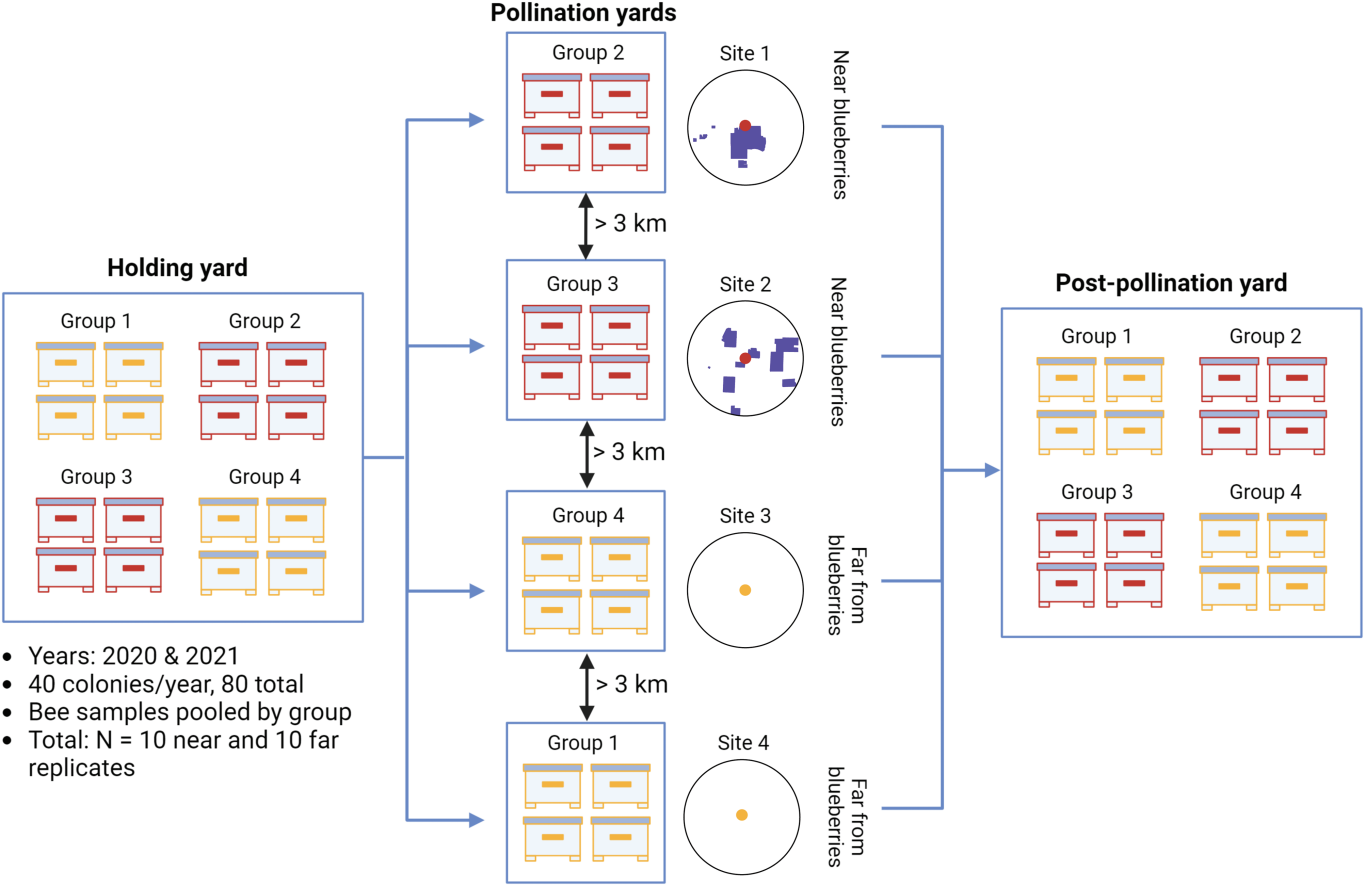
Example schematic of yard composition. Colonies at holding yards were assigned to either travel to pollination yards adjacent to highbush blueberry fields (“near” yards) or far from blueberry fields (“far” yards) in a balanced random design. Once assigned to a group, colonies within the same group traveled together to their respective yards. Holding yards and postpollination yards contained equal numbers of colonies destined to or arriving from near and far pollination yards. All near/far yards were at least 3 km apart except in 1 instance when 2 near yards were 2 km apart, and 1 instance where 1 near and 1 far yard were 2.7 km apart. Colonies in near yards were situated beside blueberry fields (<140 m away) and colonies in far yards were situated away from blueberry fields (>1,300 m away). Examples of actual sites are shown, with blueberry field indicated as shaded polygons. Dots represent site locations and circles represent a 1.5 km radius. Proximity and blueberry area data for all sites are included in Table 2.

Colonies destined to or coming from near and far pollination yards made up a balanced population in the holding yards and postpollination yards, such that there was no bias in pre- and postpollination location between colonies assigned to near and far site types (illustrated in Fig. 2). For example, in 2020, holding yards were comprised of either 8 or 16 colonies, and before the experiment began, half of the colonies at each holding yard were randomly assigned to travel to 1 (for holding yards with 8 colonies) or 2 (for holding yards with 16 colonies) of 5 different sites near highbush blueberry fields and the other half were randomly assigned to 1 or 2 of 5 sites far from highbush blueberry fields. At this time, colonies were also assigned to 4-colony groups which traveled together when moved between yards (samples of bees from each colony within the same group were later pooled into a single sample, described below). All colonies were moved to pollination yards on the same day where possible, or within a maximum of 24 h of each other when the blueberry bloom reached approximately 5%–10% (as is conventional for pollinating this crop). See Table 1 for a timeline of sampling and moving events. In both years, colonies were then relocated to postpollination yards, again with colonies belonging to the same 4-colony group traveling together and with postpollination yards receiving a balanced number of colonies coming from sites near and far from blueberries. In cases where some colonies remained at the same location for consecutive sampling events but other colonies were moved, a “sham” movement procedure was followed, such that all colonies were treated as if they were moving (i.e., they were screened, strapped, and driven in the back of a vehicle for similar durations), except that the colonies remaining in place were dropped off at the same location at which they were previously.

### Sampling Methods

At each time point, adult bees were sampled from an open brood frame into a 50 ml conical tube, placed immediately on dry ice, and transported to the University of British Columbia laboratory, where they were stored at −70 °C until all samples were collected. Bees were also sampled into a urine vial (~120 ml, with an average of 260 bees per sample), which was then filled with 70% ethanol for conducting mite counts by the alcohol wash method as previously described (Borba et al. 2022). At the same time, approximately 4 g of fresh (chalky), stored pollen was collected from comb cells (minimum of 2 patches from 2 different frames) using a wooden spatula. As these samples are part of a larger collaboration between Canadian research institutions, samples for pathogen and pollen analysis were shipped on dry ice to York University (Toronto, ON, Canada), where they were centrally stored at −70 °C, then bee samples were shipped to National Bee Diagnostic Center (NBDC; Beaverlodge, AB, Canada) and pollen samples were analyzed by Genome Quebec (described under the “pollen metabarcoding” section). Before submission to the NBDC for pathogen analysis, bee samples were pooled according to predetermined groups to create 2 composite samples per group (15 bees per colony, 60 bees in total in each sample). Pollen samples were pooled in the same manner. The rationale for this pooling strategy was to minimize sample-to-sample variation while also reducing the number of samples analyzed, as this experiment was part of a broader multiprovince effort to assess hundreds of colonies in different pollination contexts. The final sample size was therefore *n* = 5 near and *n* = 5 far replicates per year (*n* = 10 near and 10 far in total), where each replicate is represented by a pool of bees from 4 colonies that traveled together from holding yards, to near or far yards, to postpollination yards.

### Pathogen Detection

From the pooled samples described above, the NBDC conducted pathogen testing of Israeli acute paralysis virus (IAPV), deformed wing virus A (DWV-A), varroa destructor virus (VDV; also known as deformed wing virus B or DWV-B), acute bee paralysis virus (ABPV), Kashmir bee virus (KBV), chronic bee paralysis virus (CBPV), black queen cell virus (BQCV), SBV, *M. plutonius, V. apis* and *V. ceranae* (Tokarev et al. 2020). All pathogen data are reported in Supplementary Data 1.

For *V. ceranae*, *V. apis*, and *M. plutonius* detection, DNA was extracted from a 200 µl aliquot of the homogenized sample described above and analyzed by endpoint PCR. The samples were pelleted by centrifugation, the liquid was aspirated, and the pellet was allowed to dry at room temperature. Genomic (g)DNA was extracted and purified using the NucleoSpinTissue kit following the manufacturer’s instructions (Macherey-Nagel Gmbh & Co. KG, Düren, Germany). Primers used for the detection of all pathogens are found in Supplementary Data 2. PCR amplification assays used AccuStart II PCR Supermix (Quanta Bioscience, USA) and all assays were performed using 60 ng of gDNA in an Applied Bioscience Veriti 96-well Thermal Cycler (Applied Bioscience, Singapore). PCR conditions were 5 min at 95 °C for initial denaturation/enzyme activation followed by 35 cycles of 1 min at 94 °C, 1 min at 58 °C, and 1 min at 72 °C, with a final extension of 10 min at 72 °C. Amplicons were visualized by gel electrophoresis.

All viruses were detected and quantified using RT-qPCR following sample preparation methods as previously described (Borba et al. 2022). Briefly, total RNA was extracted from the second pooled sample of 60 bees using a NucleoSpinRNA kit (Macherey-Nagel Gmbh & Co.), cDNA was synthesized from 800 ng total RNA using the iScript cDNA synthesis kit (Bio-Rad Laboratories), and reactions were assembled with sSoAdvanced Universal SYBR Green Supermix (Bio-Rad Laboratories). Each virus was tested in triplicate for each sample using 3.75% of the cDNA reaction product, and absolute quantitation was performed by comparing sample values against a standard curve (generated from serially diluted, amplicon-containing plasmids). PCR conditions were 3 min at 95 °C for initial denaturation/enzyme activation followed by 40 cycles of 10 s at 95 °C and 30 s at 60 °C (except IAPV, where annealing/extension was 45 s at 60 °C). Specificity was checked by performing a melt-curve analysis (65–95 °C with increments of 0.5 °C and 2 s/step). Results were analyzed with the CFX Manager Software and exported.

### Multivariate Pathogen Analysis

Statistical analyses were conducted in R (version 4.3.0) using R Studio (version 2023.09.1 + 494) (R Core Team 2023). To determine if exposure to blueberry fields affected the composite pathogen profiles over time, we conducted a PERMANOVA analysis using the adonis2 function within the R package vegan (version 2.6-4) (Oksanen et al. 2022). The similarity was calculated using the Jaccard index since some of the pathogens (*V. ceranae*, *V. apis*, and *M. plutonius*) are limited to a presence/absence data type. We first evaluated the pathogens at time point 1 to determine if the multivariate pathogen structure among colonies destined to be distributed to different site types was indeed similar. This model included a pathogen matrix of 12 response variables (9 viruses, *M. plutonius*, *V. ceranae*, and *V. apis*), and explanatory variables of site type (levels: near and far), and year (levels: 2020 and 2021). Varroa load was initially considered in the model but was dropped as it was not a significant explanatory factor. Evaluation of the distance matrix using the “betadisper” function within the vegan package confirmed that group dispersions were homogeneous. The pathogen data and sample metadata for all time points are available in Supplementary Data 1.

After confirming that pathogens in samples from colonies destined to travel to their respective “near” and “far” site types were indeed similar before being moved, we tested for effects of blueberry proximity on multivariate pathogen structure for the remaining time points. This model included the same 12 response variables and explanatory factors site type (levels: near and far), and time (levels: t2, t3, and t4), as well as their interaction. We conducted a restricted permutation test, which only allows samples to be permuted within pooling groups to account for repeated measures over time. Varroa load was again initially considered but dropped from the final model as it was not a significant explanatory factor. Group dispersions were gain determined to be homogeneous, as described above. Because an interactive effect between site type and time point was identified, we conducted post hoc tests at each time point individually to identify the time point(s) driving the interaction, again using a reductive modeling approach similar to the examples above.

### Visualization of Pathogen Data and Assessment of Specific Pathogens

To visualize the multivariate data at each time point, we used the metaMDS function (package: vegan, version 2.6-4; specifying *k* = 2, trymax = 500, and distance = “jaccard”) (Oksanen et al. 2022) and plotted the resulting scores using ggplot2 (Wickham 2016). Differences in individual pathogen detections at t4 were also evaluated statistically using a generalized linear model (package: stats, base R) (R Core Team 2023) with a binomial vector of pathogen presence/absence as the response variable, site type and year as fixed factors, and a binomial distribution specified. Here and subsequently, we ensured appropriateness of fit by checking simulated residual plots, as enabled by the package DHARMa (version 0.4.6) (Hartig 2022).

*M. plutonius*, SBV detections, and *Varroa* loads were also analyzed over time using a generalized linear mixed effects model (*M. plutonius* and SBV) and a linear mixed effects model (*Varroa*) (package: lme4, version 1.1-33) (Bates et al. 2015). For *M. plutonius* and SBV, data were first modeled with the respective binomial presence/absence vector as the response variable, year as a fixed factor, site type and time point as an interactive term, and pooled sampling unit as a random intercept term. In both cases, the year was not influential and was dropped from the final model. Summary statistics for main effects (Type II Wald *χ*^2^ tests)were extracted using the Anova function (package: car, version 3.1-2) (Fox and Weisberg 2019). *Varroa* loads (mites per 100 bees) were averaged within pooled sampling groups to achieve the same data structure as all other pathogens/parasites, then were modeled according to the same parameters as *M. plutonius* and SBV, except a Gaussian distribution was used. No data transformation was necessary as inspection of simulated residual distributions plotted against model predictions showed no significant outliers nor heteroscedastic tendencies.

Some viruses (LSV, DWV-B, and IAPV) had a sufficient number of detections to model how viral copies per bee changed over time. Selecting only nonzero data, we log_10_-transformed the copy number and modeled the data with a linear mixed effects model, including year as a fixed effect, a site type × time point interaction term, and pooled sampling unit as a random intercept term.

### Pollen Metabarcoding

Pollen metabarcoding methods follow previously described methods (Wizenberg et al. 2023). Briefly, we employed a multilocus metabarcoding approach using ITS2 and rbcL sequences on pollen samples from time points t2 and t3, when groups were located at their respective near and far yards. We first extracted DNA using a NucleoMag DNA Food kit (Macherey-Nagel, Düren, Germany), then amplified the region of interest (PCR1), extended the length of the amplified region via read priming (PCR2), and indexed each library with a unique combination of forward and reverse primers (PCR3). We then normalized the pooled library using a SequalPrep Normalization kit (Invitrogen, Burlington, ON, Canada) and sequenced it at Genome Quebec (Illumina MiSeq PE250).

All data processing was completed in Python (v. 3.10.7), and R (v. 4.2.1) (R Core Team 2023), using the dada2 (v. 1.16.0) (Callahan et al. 2016) and purrr (v. 0.3.4) (Wickham and Henry 2023) packages. We processed returned sequence data by first trimming primers, pairing forward and reverse reads from each sample, filtering out low-quality reads and sequencing errors, and then grouping identical sequences under unique ASV’s (amplicon sequence variants). We then built a database that linked species to unique sequences associated with each primer using the MetaCurator method (Richardson et al. 2020). We used our MetaCurator libraries to parse through returned sequence data and identify the species associated with each ASV, setting a precursory condition of >0.95 similarity. After identifying the plant species associated with each sequence, we consolidated classifications at the genus level and prepared data for filtering. To control for mis-tagging during sequencing, we utilized a previously published filtering method (Richardson 2022). We used negative controls as indicators of mis-tagging frequency and filtered real sample data to remove detections with a high likelihood of representing mis-tag-associated false detections. For a complete description of our laboratory methods and bioinformatics approach, please see Wizenberg et al. (2023).

To control for within-sample sequence yield variation and its impact on the dietary richness and diversity, we rarefied the resulting matrix down to the lowest sequence hit value for each respective marker, using the rrarefy() function included in the vegan (v. 2.6-4) package (Oksanen et al. 2022) (8424 for ITS2, 19792 for rbcL). We estimated dietary richness as the number of unique genera in each pollen sample and dietary diversity using Shannon’s index, generated via the diversity() function included in the vegan package. We visualized dietary composition using the ggplot() function included in the ggplot2 (v. 4.2.0) (Wickham 2016) package. Differences in pollen diversity at t2 and t3 were assessed using a linear mixed model with time point and site type as interactive terms, year as a fixed effect, and pooled sampling unit as a random intercept term. As before, the appropriateness of fit was assessed using tools within the DHARMa package. Proportional abundance of *Vaccinium* pollen was also assessed in near and far site types using a Mann–Whitney test due to heteroskedasticity of the data (time points and years tested separately). Rarefied data are included in Supplementary Data 3.

## Results

### Pathogen Community Analysis

Including samples at all time points, LSV was both the most abundant and most prevalent virus (detected in 92.5% of samples), followed by DWV-B (88.8%), IAPV (87.5%), SBV (71.3%), DWV-A (68.8%), BQCV (65.0%), KBV (32.5%), CBPV (10.0%), and ABPV (2.5%). *V. ceranae* was also highly prevalent (detected in 100% of samples), whereas *V. apis* was detected in 32.0% of samples, and *M. plutonius* was detected in 75.0% of samples. These data are shown in Supplementary Fig. S1. Irrespective of whether colonies traveled to near or far site types, LSV loads increased over time (*χ*^2^ = 41, *df* = 1, *P* < 0.001) from a median of 19.0 million copies per bee at t1 to 278 million copies per bee at t4. All other viral loads either did not change over time (IAPV, DWV-A, and DWV-B) or a well-fitting model could not be produced due to too many missing values and problematic data distributions (SBV, BQCV, KBV, CBPV, and ABPV).

**Fig. 3.**
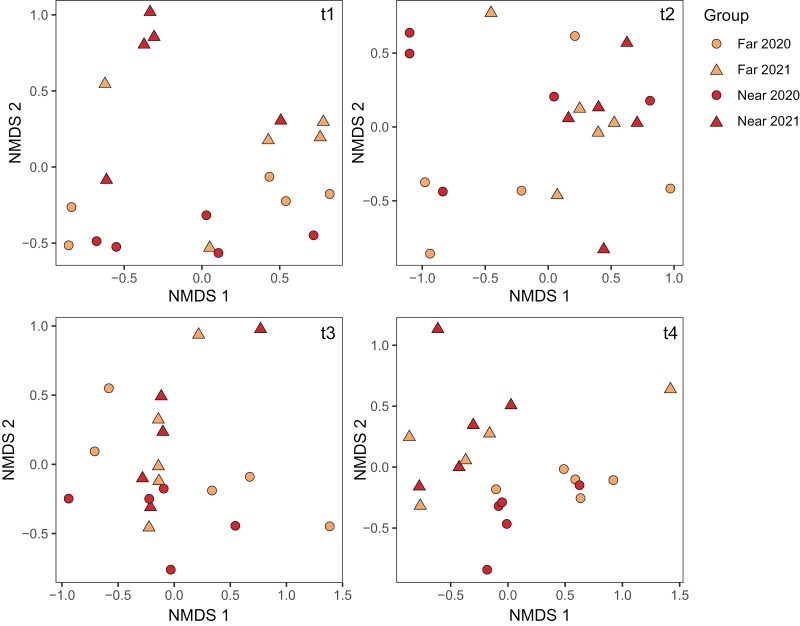
Nondimensional scaling plots illustrating honey bee pathogen matrices. Overall differences in multivariate pathogen profiles before (t1), during (t2), after (t3), and at the end (t4) of highbush blueberry pollination were evaluated using a PERMANOVA (Jaccard index) with site type (levels: far and near) and time point (levels: t2, t3, and t4) as interactive effects, year (levels: 2020 and 2021) as a fixed factor, and replicate as a blocking factor to account for repeated measures. Data originated from *N* = 20 replicates (each representing a pooled sample from 4 colonies) distributed across site types and years. Post hoc PERMANOVA tests at each time point show that the effects are driven by t4, in particular, the separation of near and far groups from 2020.

Due to the sparsity of data for most viruses, large number of pathogens measured, and presence of some pathogens limited to binomial detections (*V. ceranae, V. apis*, and *M. plutonius*), we evaluated composite pathogen profiles using a combination of nonmetric multidimensional scaling (NMDS) plots and PERMANOVA analyses with the Jaccard index for distance calculation (which considers only whether a community member was observed or not, irrespective of magnitudes). NMDS plots of the pathogen profiles at each time point showing that data points appear to cluster most strongly by year (Fig. 3). This appears to be driven mainly by differences in BQCV (which was observed in 100% of samples in 2020 but only 30% of samples in 2021), ABPV (observed in 2.5% of samples in 2020 and 17.5% of samples in 2021), and *V. apis* (observed in 47.5% of samples in 2020 and 12.5% of samples in 2021), among other minor contributors (Supplementary Fig. S1).

To investigate multivariate profiles with respect to near and far site types, we first checked if profiles in replicates destined to be moved to pollination yards (*n* = 5 in each year, representing 80 contributing colonies) were similar at t1, before being moved to sites near and far from highbush blueberries. As expected, found no significant differences (PERMANOVA, index = Jaccard; Site type: *F* = 0.89; df = 1, 17; *P* = 0.54; Year: *F* = 1.4; *df* = 1, 17; *P* = 0.18). Evaluating profiles at t2, t3, and t4 together, we identified a significant interactive effect of site type and time point (PERMANOVA, index = Jaccard; Interaction: *F* = 1.7; *df* = 2, 53; *P* = 0.021) as well as significant main effects of site type (*F* = 1.4; *df* = 1, 53; *P* = 0.013), time point (*F* = 1.6, *df* = 2, 53; *P* = 0.031), and year (*F* = 2.1; *df* = 1, 53; *P* = 0.021). Post hoc comparisons within each time point show that the site type effects are driven by differences at t4, the only individual time point for which pathogen profiles are significantly linked to site type (*F* = 2.2; *df* = 1, 17; *P* = 0.028).

**Fig. 4.**
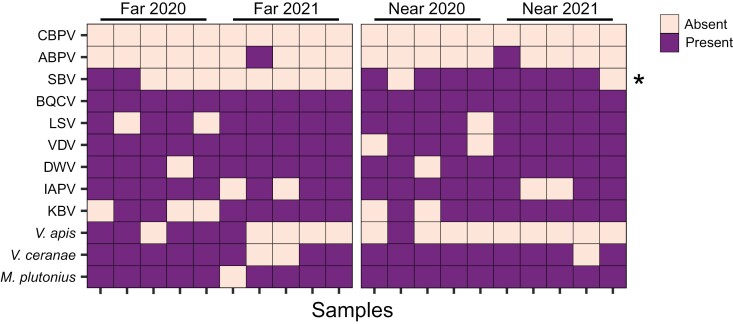
Pathogen detections at time point t4. Pooled samples (4 colonies per sample, *n* = 5 samples per condition, per year) were taken approximately 6 wk after the beginning of the highbush blueberry pollination period. All pathogens were detected with qPCR or PCR. CBPV, chronic bee paralysis virus; ABPV,acute bee paralysis virus; KBV, Kashmir bee virus; IAPV, Israeli acute paralysis virus; VDV, varroa destructor virus (also known as DWV-B); SBV, sacbrood virus; LSV, Lake Sinai virus; BQCV, black queen cell virus. Asterisks indicate significant differences determined by logistic regression with site type and year as fixed factors. BQCV was present in all samples at t4 and thus not included in post hoc comparisons, but is still visualized here. Grey tiles indicate missing data.

Next, we evaluated detections of each pathogen at t4 to determine what individual profiles were driving the site type effect (logistic regression; fixed factors: site type and year) (Fig. 4). SBV was the pathogen most significantly linked to site type (near vs. far) (*χ*^2^ = 8.2; *df* = 1; *P* = 0.0042; *α*/*n* = 0.0045 for Bonferroni correction), with higher frequency of detections in the samples near highbush blueberries. The next leading pathogen linked to site type was *V. apis*, but differences were not significant with Bonferroni correction (*χ*^2^ = 5.2; *df* = 1; *P* = 0.022; *α*/*n* = 0.0045).

### *Varroa* Loads

We analyzed *Varroa* loads over time using a linear mixed model and found no effect of year, site type, time point, or site type × time point interaction (Table 3). In a preliminary analysis, we also included *Varroa* loads as a covariate in our multivariate community analysis described above (see *Materials and Methods* section), but it was not a significant predictor and was thus removed from the model. *Varroa* loads, therefore, appear not to be linked to our experimental parameters and do not seem to be influential for profiles of other pathogens in this study.

**Table 3.** Statistical analysis of *Varroa* loads

Analysis of deviance table (Type II Wald *χ*^2^)	*χ* ^2^	*df*	*P*
Year (factor levels: 2020, 2021)	0.28	1	0.59
Time point (continuous)	1.3	1	0.25
Site type (factor levels: near, far)	1.2	1	0.27
Time point × Site type	0.38	1	0.54

Random effect	Variance	SD	Levels
Random intercept: [Pooled group]	0.15	0.39	20

Full model *R*^2^ = 0.48.

Formula = Varroa load (percent) ~ Year + Site type × Time point + (1|Pooled group).

Family = Gaussian.

Observations = 79.

### Assessment of *M. plutonius* and SBV Over Time

**Fig. 5.**
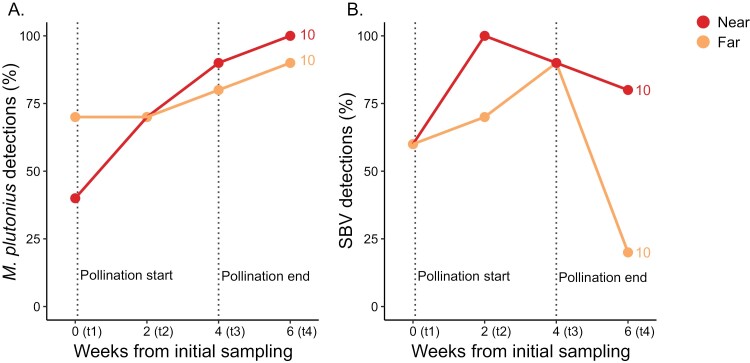
Percent prevalence of *M. plutonius* and SBV at near vs. far sites over time. A) *M. plutonius* and B) SBV percent prevalence in replicates near and far (*n* = 10 each) from highbush blueberries. There was no effect of year for these pathogens so data from 2020 and 2021 are pooled to show overall trends. Numbers above the dots indicate replicates, each representing a pooled sample from 4 colonies. *M. plutonius* detections are significantly linked to the time point (*χ*^2^ = 6.18, *P* = 0.013), but no other significant relationships were identified (logistic regression, factors: site type, time point, and their interaction). Weeks from initial sampling are approximate and correspond to 14, 28, and 44 days in 2020 and 17, 28, and 40 days in 2021.

While we did not identify a significant effect of site type at any time point except t4 in the multivariate pathogen analysis, we were still interested in patterns of *M. plutonius* and SBV detections over time, specifically. We found that *M. plutonius* detections increased over time (*χ*^2^ = 6.18; *df* = 3; *P* = 0.013), with no effect of site type (*χ*^2^ = 0.14; *df* = 1; *P* = 0.71). Additionally, although detections in near samples did tend to increase at a faster rate than far samples (*M. plutonius* detection frequency increases from 40% to 100% over the 6-wk period in the near group, compared to 70%–90% in the far group over the same time period), the site type-by-time point interaction term was marginally not significant (*χ*^2^ = 3.04; *df* = 1; *P* = 0.081; generalized linear model; factors: site type, time point, and their interaction; random variable: pooled sampling unit) (Fig. 5). Using the same model structure, we found that SBV detections were marginally not significantly linked to site type (*χ*^2^ = 2.98; *df* = 1; *P* = 0.084), time point (*χ*^2^ = 0.14; *df* = 1; *P* = 0.71) or their interaction (*χ*^2^ = 2.54; *df* = 1; *P* = 0.11). The significant effect of site type identified at t4 is, therefore, not sufficiently strong to drive an effect in this larger model. Since near and far groups had the same, high SBV detection frequencies at t3 (9/10 samples), the difference observed at t4 appears to be a result of disproportionate clearance of the virus in far group colonies.

### Pollen Analysis

**Fig. 6.**
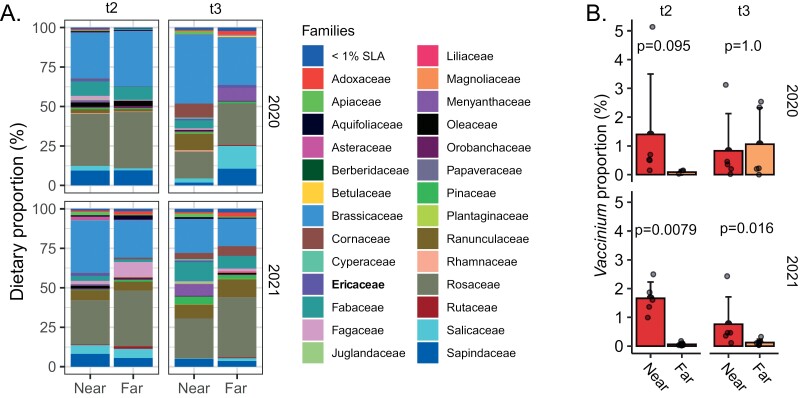
Pollen diversity and *Vaccinium* pollen abundance collected at near and far pollination yards. A) Family diversity did not differ between near and far yards nor time points but did significantly differ between years (higher in 2021). Ericaceae, the family to which *Vaccinium* belongs, is shown in bold. B) *Vaccinium* pollen proportional abundance was evaluated during the pollination period and tended to be higher at near yards (Mann–Whitney tests).

Diversity of pollen collected after colonies spent at least 2 wk at their pollination yards (t2 and t3 sampling) was overall similar between near and far site types (Fig. 6A), but proportional *Vaccinium* abundance tended to be higher at near sites (Fig. 6B). Pollen samples were dominated by species in the Brassicaceae and Rosaceae families at all time points in both years. Pollen diversities at t2 and t3 were significantly higher in 2021 than 2020 (*χ*^2^ = 5.3, *df* = 1, *P* = 0.02) but did not differ by time point (*χ*^2^ = 0.27, *df* = 1, *P* = 0.61) nor between near/far site types (*χ*^2^ = 1.8, *df* = 1, *P* = 0.18). *Vaccinium* proportional abundance was significantly higher at near sites for t2 samples (Mann–Whitney test; *W* = 25, *P* = 0.0079) and t3 (*W* = 24, *P* = 0.016) in 2021 but was not significantly different at either time point in 2020 (though it again tended to be higher at near sites for t2 samples) (t2: *W* = 10, *P* = 0.095; t3: *W* = 13, *P* = 1.0).

## Discussion

There has been an ongoing concern among beekeepers in British Columbia and elsewhere in North America that engaging in highbush blueberry pollination may lead to a higher prevalence of EFB disease in their colonies (Wardell 1982, Higo et al. 2019). In this study, we evaluated *M. plutonius* detections as well as 9 viruses and 2 microsporidian parasites in honey bee colonies located far from and near to highbush blueberry fields. In a top-down approach, we analyzed all pathogen and parasite variables together (PERMANOVA, Jaccard index) and identified a significant interaction between blueberry exposure and time points. Post hoc testing showed that this interaction was driven by delayed differences at t4, and analysis of individual pathogens revealed SBV, not *M. plutonius*, to be the main driver of this pattern.

Our data are consistent with Fowler et al. (2023), who found no relationship between EFB prevalence and blueberry pollination in Michigan. We found no significant interaction between *M. plutonius* detections and time (Fig. 5) but detections tended to increase faster in the colonies located far from blueberries compared to those near blueberries. It is possible that if the experiment were extended for another 2 wk, we may have detected a significant, though very delayed, effect. Investigating patterns of SBV over time, we found that at the end of the pollination period, both near and far groups had the same SBV detection frequency (90% of colonies tested positive), but at t4, the far group dropped to 20% while the near group remained high at 80%. This suggests that the differences observed at t4 are due to far colonies disproportionately recovering from prior SBV infections compared to near colonies. Some potential mechanisms underlying these patterns are hereupon discussed.

Like other honey bee viruses, SBV can spread between colonies as a result of drifting, robbing, and possibly through contact between bees at forage sources (Chen et al. 2006, Alger et al. 2019). The latter 2 of these mechanisms could be amplified to some degree when colonies are moved into pollination yards, as forage availability and the population of proximal colonies changes. However, the near and far colonies in this study had the same number of SBV detections at t3, the end of the pollination period, so the difference we found at t4 is not driven by dispersal. Previous research has identified a link between fungicide exposure and increased susceptibility to some viral infections (Degrandi-Hoffman et al. 2015, O’Neal et al. 2019); for example, Degrandi-Hoffman et al. (2015) found that boscalid and pyraclostrobin exposure affected DWV levels. We did not analyze agrochemical data here, but this concept offers precedent for how some viruses may be more affected than others by crop exposure. To our knowledge, interactions between SBV infections and agrochemical exposure have not yet been mechanistically investigated, but it is possible that the colonies near highbush blueberry fields were less able to clear existing SBV infections due to differences in prior agrochemical exposure. Indeed, in a larger nationwide study, which included some data presented here, SBV interacts with numerous other stressors, including several agrochemicals (French et al. 2024). Curiously, a positive association between SBV and the presentation of EFB disease was recently reported, but the causal direction and mechanism underlying this relationship are not yet known. We investigated *M. plutonius* and not EFB disease, and our data do not show a positive relationship between these factors, but the concept of a relationship between these 2 brood afflictions is intriguing and bears further investigation.

Within colonies, SBV is transmitted horizontally (between adult workers through trophallaxis, for example), vertically (from queen to offspring via eggs), and diagonally (from nurse to larva during feeding, or larva to nurse during hygienic behavior) (Chen et al. 2006, Wei et al. 2022). As with other viruses, *V. destructor* mites act as a vector, and we were somewhat surprised to detect no relationship between mite loads and SBV detections. However, previously reported correlations are, while significant, not strong (with Pearson coefficients of 0.17 and 0.24, for example) (Borba et al. 2022). We speculate that our pooled sampling approach, smaller sample sizes, and differences in seasonal timing of samples compared to Borba et al. (2022) contribute to our observed lack of relationship between mite loads and SBV. Additionally, the average mite load across all our samples was only 0.72% (0.65% in 2020 and 0.78% in 2021), which is likely below the level at which an association with viruses would be observed.

While our experimental design allowed us to investigate patterns of pathogen prevalence in relation to the regional timing of blueberry pollination, it does have limitations. As noted, 1 limitation in this study is that our statistical power was low. While the effective sample size is relatively small (*n* = 5 unique replicates per year, per site type), since each replicate represents a pooled sample of 4 colonies, this means that a total of 80 colonies participated in this study across years. Despite this limitation, the magnitude of the SBV effect was still large enough to detect at t4. We and others have anecdotally observed that colony health tends to decline around this time point after engaging in blueberry pollination, and we argue that SBV might be an underappreciated pathogen contributing to this observation.

A second limitation is that the high prevalence of highbush blueberry fields in the region limited our ability to place colonies in the far group such that highbush blueberry fields were completely outside of the foraging range. In our case, we chose the near and far field sites we did for 2 main reasons: (i) blueberry occurrence in the study location is so high that finding field sites completely outside of longer foraging radii (e.g., where 95% of foraging activity would take place) was impractical and (ii) the farther the distance between sites, the larger the differences in other variables would be (e.g., other landscape and land use parameters, microclimates, density of other beehives, etc.). Unfortunately, some contact with blueberries did occur at our far sites: Although the fraction of *Vaccinium* pollen in colonies at near sites was higher than far sites, some *Vaccinium* pollen was still detected at far sites, particularly at t3 in 2020. The dominant native *Vaccinium* in the study area (red huckleberry, *Vaccinium parvifolium*) may have contributed to this pattern, as there is some overlap in bloom timing with highbush blueberries, but it is not a typical forage source for honey bees. Overall, we argue that the chosen sites strike a balance between proximity to blueberries and minimizing extraneous variables. Moreover, the near sites have the key difference of being located immediately next to blueberry fields, as opposed to far sites, which were, by definition, farther away.

In this study, we did not measure the prevalence of EFB disease symptoms in colonies, which is distinct from detections of *M. plutonius*, as testing positive for *M. plutonius* does not necessarily mean that the colony is symptomatic (Milbrath et al. 2021). Additionally, since larval samples were not part of this experiment, we are unable to determine to what extent SBV may or may not contribute to disease manifestation or appearance. Future experiments investigating *M. plutonius* and blueberry pollination should include SBV analysis of symptomatic and asymptomatic larvae to better understand the possible relationship between these 2 pathogens (as well as LSV) and disease presentation, as there is some degree of overlap in their symptoms and prior evidence for interactions.

Like EFB, SBV symptoms are thought to occur most frequently in the spring (Bailey 1969), and like EFB and American foulbrood (AFB), dried SBV-infected larvae can also have a scale-like appearance and larvae may die after cell capping, which can lead to a similar presentation of spotty brood patterns and perforated cell caps (Grabensteiner et al. 2001, Milbrath 2021, Milbrath et al. 2021). But unlike these 2 bacterial diseases, SBV can replicate in adult bees and decrease their lifespan (Wang and Mofller 1970, Bailey and Fernando 1972). SBV is highly prevalent in Canada (National Bee Diagnostic Center 2017), and SBV levels in adult bees sampled in fall are associated with smaller fall and spring cluster sizes (Borba et al. 2022) as well as increased winter mortality of colonies (Desai and Currie 2016). This may, in part, explain the delayed appearance of site-type effects in honey bee colonies pollinating blueberries if cascading effects of shorter-lived adults are influencing susceptibility to subsequent opportunistic pathogens.

All this is not to say that SBV detections are higher as a result of blueberry pollination, specifically. Stressors affecting disease prevalence may also originate not only from the pollinated crop but also from surrounding landscapes or interactions with other pollinators. Indeed, pesticide risk associated with blueberry pollination may not be driven by the crop itself but by other crops present in the area (Graham et al. 2022). We speculate that there could be a broader effect of agricultural landscape exposure in general, the influence of which may or may not manifest depending on the presence or absence of additional extraneous variables. Honey bee colonies near-blueberry fields may also experience different or more frequent interactions with other pollinators, wild or managed, attracted to the crop and nearby forage, leading to different disease profiles postpollination. Importantly, we found that the diversity of foraged pollen and the dominant foraged genera at near and far sites were overall similar, suggesting that nonblueberry dietary differences are unlikely to have substantially influenced our results. More work is necessary to replicate the association we find here and to determine the mechanism by which it manifests.

SBV, amongst a plethora of other stressors, is generally not considered to be a major concern for honey bee health in North America. However, our data suggest that it may be an underappreciated pathogen. Relatively little research has been conducted on SBV relative to, e.g., *M. plutonius*, *Paenibacillus larvae* (the agent causing AFB disease), *Vairimorpha* spp., and DWV. Given that we show significant associations between SBV detections and highbush blueberry exposure, our findings suggest that this is an agriculturally relevant virus that deserves further attention.

## Supplementary Material

toae119_suppl_Supplementary_Data_1

toae119_suppl_Supplementary_Data_2

toae119_suppl_Supplementary_Data_3

toae119_suppl_Supplementary_Figure_S1

## Data Availability

All data presented in this manuscript are available in Supplementary Data 1 & 2. Supplementary Data 1 includes all pathogen and parasite data. Primer sequences for PCR detections are included in Supplementary Data 2. Supplementary Data 3 includes pollen metabarcoding data.
