# Correction to: Higher prevalence of sacbrood virus in *Apis mellifera* (Hymenoptera: Apidae) colonies after pollinating highbush blueberries

**DOI:** 10.1093/jee/toae198

**Published:** 2024-08-20

This is a correction to: Alison McAfee, Sarah K French, Sydney B Wizenberg, Laura R Newburn, Nadejda Tsvetkov, Heather Higo, Julia Common, Stephen F Pernal, Pierre Giovenazzo, Shelley E Hoover, Ernesto Guzman-Novoa, Robert W Currie, Patricia Wolf Veiga, Ida M Conflitti, Mateus Pepinelli, Lan Tran, Amro Zayed, M Marta Guarna, Leonard J Foster, Higher prevalence of sacbrood virus in *Apis mellifera* (Hymenoptera: Apidae) colonies after pollinating highbush blueberries, *Journal of Economic Entomology*, 2024;, toae119, https://doi.org/10.1093/jee/toae119

Upon original publication, several errors were present in the Abstract section.

In the first sentence, ‘(*Apis mellifera* L.)’ was incorrectly given as ‘(*Apis mellifera*) L’., as follows:

Highbush blueberry pollination depends on managed honey bees (*Apis mellifera*) L. for adequate fruit sets; however, beekeepers have raised concerns about the poor health of colonies after pollinating this crop.

Additionally, in the following sentence, 'Tokarev et al.', 'Microsporidia: Nosematidae', and 'Zander' were incorrectly italicized:

Nine viruses, as well as *M. plutonius*, *Vairimorpha ceranae*, and *V. apis* [Tokarev et al., *Microsporidia: Nosematidae*; formerly *Nosema ceranae* (Fries et al.) and *N. apis (Zander)*], were detected by PCR and compared among colonies located near and far from blueberry fields.

The Publisher apologizes for these errors, which have been corrected online.

