# Supplementary material for: Higher prevalence of sacbrood virus in Apis mellifera (Hymenoptera: Apidae) colonies after pollinating highbush blueberries
Source: J Econ Entomol. 2024 Jun 15;117(4):1324–35. doi: 10.1093/jee/toae119 (PMC11318621; doi:10.1093/jee/toae119)
Supplement: toae119_suppl_Supplementary_Figure_S1 [file toae119_suppl_supplementary_figure_s1.docx]

Higher prevalence of sacbrood virus in honey bee (*Apis mellifera*) colonies after pollinating highbush blueberries

Alison McAfee^1,2,a^, Sarah K. French^3^, Sydney Wizenberg,^3^ Laura R. Newburn,^3^ Nadejda Tsvetkov^1^, Heather Higo^1^, Julia Common^1^, Stephen F. Pernal^4^, Pierre Giovenazzo^5^, Shelley E. Hoover^6^, Ernesto Guzman-Novoa^7^, Robert W Currie^8^, Patricia Wolf Veiga^9^, Ida M. Conflitti^3^, Mateus Pepinelli^3^, Lan Tran^4^, Amro Zayed^3^, M. Marta Guarna^4,b^ and Leonard J. Foster^1,a,b^

1. Department of Biochemistry and Molecular Biology, Michael Smith Laboratories, University of British Columbia, Vancouver, British Columbia V6T1Z4, Canada
2. Department of Applied Ecology, North Carolina State University, Raleigh, NC 27695, USA
3. Department of Biology, York University, Toronto, ON M3J 1P3, Canada
4. Agriculture and Agri-Food Canada, Beaverlodge Research Farm, Beaverlodge, AB T0H 0C0, Canada
5. Département de biologie, Université Laval, Ville de Québec, QC G1V 0A6, Canada
6. Department of Biological Sciences, University of Lethbridge, Lethbridge, AB T1K 3M4, Canada
7. School of Environmental Sciences, University of Guelph, Guelph, ON N1G 2W1, Canada
8. Department of Entomology, University of Manitoba, Winnipeg, MB R3T 2N2, Canada
9. Northwestern Polytechnic, National Bee Diagnostic Centre, Beaverlodge, AB T0H 0C0, Canada

^a^Corresponding authors: [alison.n.mcafee@gmail.com](mailto:alison.n.mcafee@gmail.com), [foster@msl.ubc.ca](mailto:foster@msl.ubc.ca)

^b^Authors contributed equally


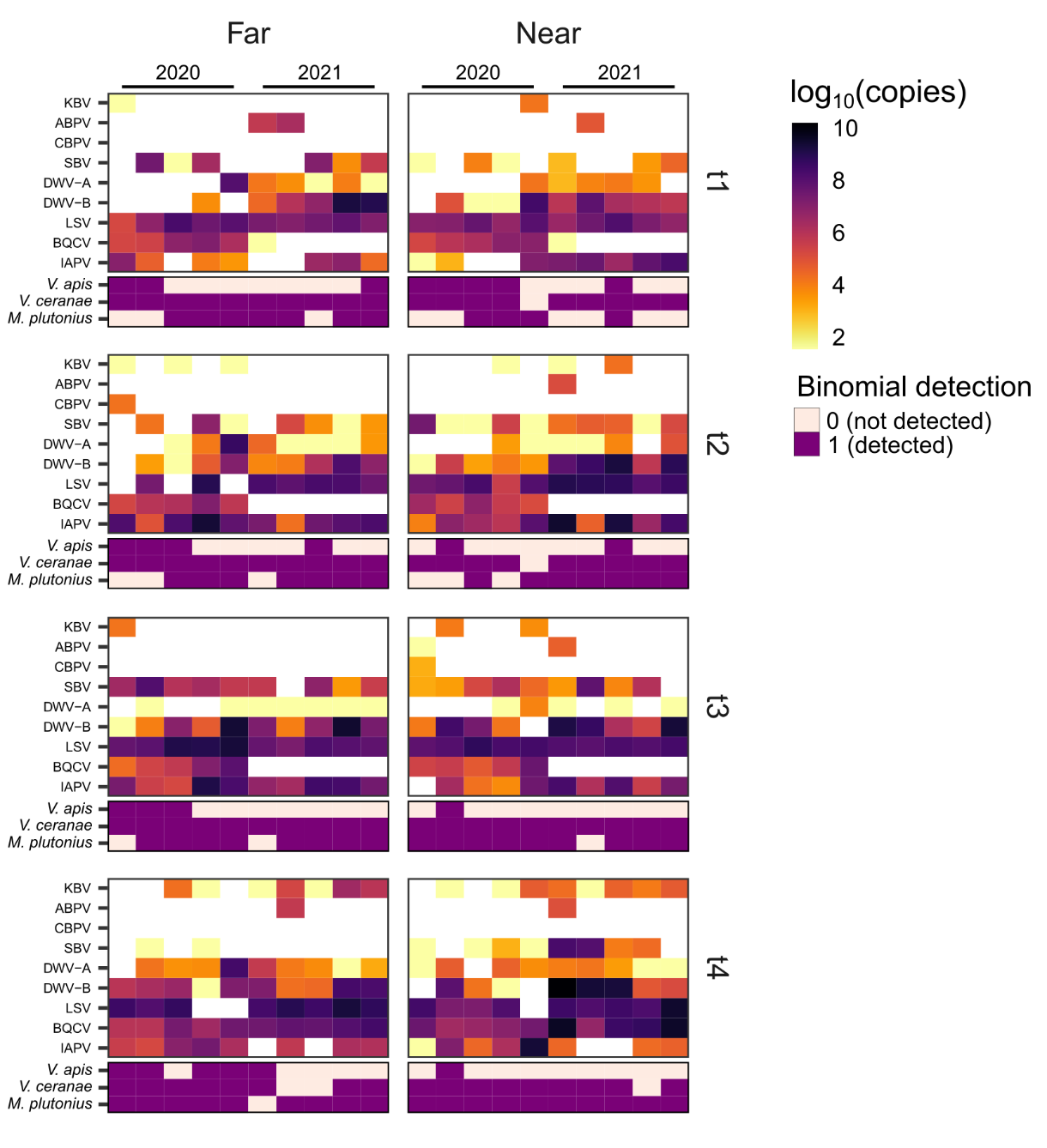


Supplementary Figure S1. Pathogen abundances and detections in all samples and time points. Viral abundances were determined by qPCR. White tiles indicate that the virus was not detected. *V. apis, V. ceranae*, and *M. plutonius* detections were determined using endpoint PCR, and their presence/absence is indicated with purple/pink tiles, respectively. Grey tiles indicate that samples could not be evaluated.
